# Malnutrition is Associated with Behavioral and Psychiatric Symptoms of Dementia in Older Women with Mild Cognitive Impairment and Early-Stage Alzheimer’s Disease

**DOI:** 10.3390/nu11081951

**Published:** 2019-08-20

**Authors:** Ai Kimura, Taiki Sugimoto, Kazuya Kitamori, Naoki Saji, Shumpei Niida, Kenji Toba, Takashi Sakurai

**Affiliations:** 1 Center for Comprehensive Care and Research on Memory Disorders, National Center for Geriatrics and Gerontology, Obu 474-8511, Japan; 2 Medical Genome Center, National Center for Geriatrics and Gerontology, Obu 474-8511, Japan; 3 Department of Cognitive and Behavioral Science, Graduate School of Medicine, Nagoya University, Nagoya 466-8550, Japan; 4 Department of Public Health, Graduate School of Health Sciences, Kobe University, Kobe 657-8501, Japan; 5 Department of Food and Nutritional Environment, College of Human Life and Environment, Kinjo Gakuin University, Nagoya 463-0021, Japan

**Keywords:** Alzheimer’s disease, mild cognitive impairment, dementia, nutritional status, BPSD

## Abstract

We examined the nutritional status and its association with behavioral psychiatric symptoms of dementia (BPSD) among 741 memory clinic patients (normal cognition (NC), 152; mild cognitive impairment (MCI), 271; early-stage Alzheimer disease (AD), 318). Nutritional status and BPSD were assessed using the Mini Nutritional Assessment Short-Form (MNA-SF) and the Dementia Behavior Disturbance Scale (DBD), respectively. Compared to subjects with NC, more subjects with MCI and early-stage AD were at risk of malnutrition (MNA-SF, 8–11: NC, 34.2%; MCI, 47.5%; early-stage AD, 53.8%) and were malnourished (MNA-SF, 0–7: NC, 4.6%; MCI, 5.9%; early-stage AD, 8.2%). Among patients with MCI or early-stage AD, those at risk of/with malnutrition showed higher DBD scores than those well-nourished (12.7 ± 9.0 vs. 9.5 ± 7.3; *p* < 0.001). Moreover, analysis of covariance adjusting for confounders showed that nutritional status was significantly associated with specific BPSD, including “verbal aggressiveness/emotional disinhibition” (F = 5.87, *p* = 0.016) and “apathy/memory impairment” (F = 15.38, *p* < 0.001), which were revealed by factor analysis of DBD. Our results suggest that malnutrition is common among older adults with mild cognitive decline, and possibility that nutritional problems are associated with individual BPSD.

## 1. Introduction

With the aging of the world’s population, the prevalence of dementia is rapidly increasing. Alzheimer’s disease (AD) is the most common type of dementia. Given that no basic medical treatment of AD is established at present, prevention of onset of AD in patients at risk of the disease, as well as care of those with AD, remains the most urgent of challenges in clinical practice.

Nutritional problems, notably weight loss, are frequently seen among patients with AD, especially those with moderate to severe AD. Nutritional problems are associated with adverse outcomes, such as rapid cognitive decline [1], a high rate of institutionalization [2], and increased mortality [3]. However, most studies investigated nutritional status among patients with moderate to severe AD. A few studies recently reported that nutritional problems, including appetite changes, weight loss, and sarcopenia, start with mild cognitive impairment (MCI) and early-stage AD [4,5,6,7]. Furthermore, a previous study reported that low body mass index (BMI) predicts progression of MCI to dementia [8]. Although the detailed mechanisms of nutritional problems in AD patients are not fully understood, nutritional problems appear to be important, albeit modifiable, factors that may affect the prognosis of dementia.

Besides cognitive and functional declines, behavioral and psychological symptoms of dementia (BPSD) represent a characteristic feature of AD, with at least one neuropsychiatric symptom seen in more than 80% of AD patients [9]. The presence of BPSD is associated with rapid cognitive decline and a high rate of mortality. Further, BPSD is strongly associated with caregiver burden [10,11] and is among the major reasons for deciding to institutionalize patients with AD [12]. BPSD vary by type and severity of dementia, with some symptoms, such as apathy, shown to increase from early stages of dementia [10,13]. Therefore, effective prevention and care strategies for BPSD from early-stage AD are necessary.

While the mechanisms of BPSD remain less well understood, several studies showed that risk factors for BPSD include patient-related (older age, gender, less education, and marital status) and disease-related (severity of disease, disease duration, and presence of the *APOE4* allele) factors [14,15]. Additionally, nutritional problems are also shown to be associated with BPSD, with Spaccavento et al. reporting a significant association between malnutrition and BPSD [16], and White et al. showing that the Neuro-psychiatric Inventory (NPI) was inversely correlated with BMI in institutionalized subjects with dementia [17]. Since these studies focused on patients with moderate to severe AD, however, little is known about the association between nutritional problems on BPSD in those with MCI and early-stage AD.

In the present study, we first aimed to clarify nutritional status of patients with MCI and early-stage AD compared to normal cognition (NC). Second, we focused on patients with MCI and early-stage AD, to examine the relationship between their nutritional status and various domains of BPSD. Clarifying the relationship between nutritional status and BPSD may provide a basis for effective preventive strategies against BPSD.

## 2. Materials and Methods

### 2.1. Subjects

The subjects of this study were outpatients (aged 65–89 years) who presented to the Memory Clinic at the National Center for Geriatrics and Gerontology (NCGG) of Japan during the period from September 2010 to January 2015. For the present analysis, we included patients with a Mini-Mental State Examination (MMSE) score of 21 or more who were diagnosed with NC, MCI, and early-stage AD, given that a MMSE score of 21 or more may be used as a surrogate measure for the Clinical Dementia Rating for staging of questionable (0.5) to mild (1.0) dementia in AD [18]. The subjects who had subjective cognitive complaints but were shown to have normal cognition on a neuropsychological assessment were diagnosed with NC. MCI and AD were diagnosed based on the criteria of the National Institute on Aging-Alzheimer’s Association Workgroups [19,20]. The study focused on female patients, given the well-known gender difference in BPSD manifestations [21]. A total of 846 patients were recruited, and, of these, 741 patients were included in analyses after excluding 105 patients with missing data. Of these, 152, 271, and 318 patients were diagnosed as having NC, MCI, and early-stage AD, respectively. The study protocol was approved by the Ethics/Conflict of Interest Committee at the NCGG. Written informed consent was obtained from all patients before their study participation.

### 2.2. Measurements

#### 2.2.1. Nutritional Status

Nutritional status was assessed by using the Mini Nutritional Assessment Short-Form (MNA-SF) [22], BMI, and serum levels of albumin and several micronutrients known to be associated with cognitive impairment (vitamin B_1_, vitamin B_12_, and folic acid) [23]. The reliability and validity of the MNA-SF have been established in subjects with dementia [24]. The MNA-SF consists of 6 questions: anthropometric measurement (BMI and weight loss), global assessment (motility), dietary questions (food intake), and health assessment (acute diseases and neurological problems). The MNA-SF was answered by the subjects’ families or caregivers. The total MNA-SF score ranges from 0 to 14, with the score of 12–14, 8–11, and 0–7 defined as being well-nourished, being at risk of malnutrition, and malnourished, respectively [25].

#### 2.2.2. Behavioral and Psychological Symptoms of Dementia

BPSD was assessed by using the Dementia Behavior Disturbance Scale (DBD) [26]. DBD consists of 28 items, and the frequency of each item was rated on a scale of 0–4 points (0 = never, 1 = infrequent, 2 = sometimes, 3 = frequent, and 4 = always) by the subjects’ families or caregivers, with high scores construed to indicate a greater severity of BPSD. The DBD includes various domains of behavioral disturbance, such as passivity, agitation, eating disturbances, aggressiveness, diurnal rhythm disturbances, and sexual disinhibition. The reliability and validity of the Japanese version of the DBD have previously been established [27].

#### 2.2.3. Other Assessments

Demographic data on the subjects’ age, their years of education, marital status (never married, married, divorced, or widowed), smoking status, drinking status, financial condition, and living situation were obtained from their caregivers using a questionnaire. The subjects were assessed for the presence of chronic disease (diabetes mellitus, hypertension, dyslipidemia, cardiac disease, and stroke) and the number of medications they were on. Polypharmacy was defined as five or more regularly prescribed drugs [28]. Global cognitive function was examined by using the MMSE, with the scores ranging from 0 to 30 [29]. Basic activities of daily living (BADL) and instrumental ADL (IADL) were evaluated by using the Barthel index (BI) [30] and the Lawton index (LI) [31], respectively. Vitality was assessed by using the vitality index (VI) [32]. This questionnaire included five items (waking pattern, communication, feeding, on and off toilet, and rehabilitation and other activities) and was answered by the subjects’ families or caregivers. With the total score of this questionnaire ranging between 0 and 10 points, it is judged that the closer the score comes to 0 points, the less the vitality. All subjects were evaluated for depressive mood by using the 15-item Geriatric Depression Scale (GDS-15) [33]. The GDS-15 has been shown to have acceptable sensitivity and specificity when used with people with mild to moderate dementia [34].

### 2.3. Statistical Analysis

Differences in the subjects’ characteristics were analyzed at the respective cognitive stages (NC, MCI, and early-stage AD) by using the Kruskal-Wallis rank test with Dunn’s multiple comparisons post hoc test for continuous variables, and a chi-squared test for categorical variables. The prevalence of well-nourished patients (MNA-SF score of 12–14), those at risk of malnutrition (MNA-SF score of 8–11), and those with malnutrition (MNA-SF score of 0–7) was calculated for respective cognitive stages and compared by using the chi-squared test.

In order to examine the association between nutritional status and BPSD in patients with cognitive impairment, we focused on those with MCI and early-stage AD. Since the state of MCI and early-stage AD are seamless and are difficult to distinguish clinically in a precise sense, we combined them in the analyses. In this population, those at risk of malnutrition and those with malnutrition were analyzed as one group (MNA-SF score of 0–11). First, differences in characteristics between the well-nourished subjects and those at risk of/with malnutrition were analyzed using the Mann-Whitney U test for continuous variables and the chi-squared test for categorical variables. Since the DBD includes various domains of BPSD, a factor analysis (principal factor method and promax rotation) was carried out on 28 subitems of the DBD. Items with a factor loading of <0.4 were deleted, and six factors were identified (Table 1) and were interpreted as “verbal aggressiveness/emotional disinhibition”, “motor aggressiveness”, “behavior disturbance”, “apathy/memory impairment”, “incontinence”, and “sexual disinhibition”. Differences in these factor scores were compared between the well-nourished subjects and those at risk of/with malnutrition by using one-way analysis of variance (ANOVA) and covariance (ANCOVA). ANCOVA was carried out using two models, with Model 1 adjusted for age and education and Model 2 adjusted for age, education, marital status, living situation, MMSE, BI, LI, VI, GDS, polypharmacy, and comorbid conditions, which are known to be associated with BPSD [14,15].

All analyses were carried out by using the Japanese version of SPSS for Windows v23.0 (IBM Corporation, Armonk, NY, USA). Values are means ± SDs unless otherwise indicated. *p* values < 0.05 were considered statistically significant.

## 3. Results

### 3.1. Nutritional Status at the Respective Cognitive Stages

**Figure 1:**
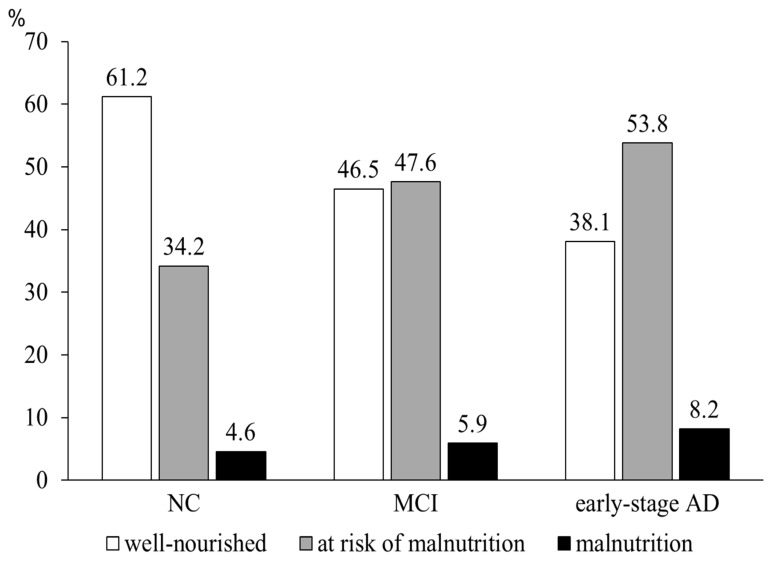
The prevalence of subjects at risk of/with malnutrition at various cognitive stages. Nutritional status was assessed by using the MAN-SF. Subjects were classified by the MNA-SF scores into well-nourished subjects (score, 12–14), those at risk of malnutrition (8–11), and those malnutrition (0–7). AD, Alzheimer’s disease; MCI, mild cognitive impairment; NC, normal cognition.

The clinical profile of the subjects at different cognitive stages is shown in Table 2. The subjects’ mean age and MMSE score were 76.6 ± 5.8 years and 25.0 ± 2.9, respectively. Their mean total MNA-SF score and BMI were 11.0 ± 2.1 and 22.0 ± 3.4 kg/m^2^, respectively. In the post hoc analyses, those with MCI and early-stage AD had significantly lower MNA-SF scores than those with NC (NC, 11.7 ± 1.9; MCI, 11.1 ± 2.1; early-stage AD, 10.7 ± 2.1; *p* < 0.001). A review of the MNA-SF showed that those with MCI and early-stage AD had decreased food intake and increased neuropsychological disturbances. The prevalence of the well-nourished subjects, those at risk of malnutrition, and those with malnutrition are shown in Figure 1. The prevalence of those at risk of malnutrition (NC, 34.2%; MCI, 47.6%; early-stage AD, 53.8%) and those with malnutrition (NC, 4.6%; MCI, 5.9%; early-stage AD, 8.2%) were higher among those with MCI and early-stage AD (*p* < 0.001).

The post hoc analyses also demonstrated that the patients with early-stage AD had significantly lower serum levels of albumin and folic acid than those with NC. The serum levels of vitamin B_1_ and B_12_ were not different (Table 2). Moreover, those with MCI and early-stage AD were more likely to be older; have fewer years of education; lower performances in cognition, IADL, and vitality; higher DBD scores; and more likely to be divorced or widowed than those with NC. Again, early-stage AD patients showed lower performances in BADL and were more likely to be living alone than those with NC.

### 3.2. Association between Nutritional Status and Behavioral Psychiatric Symptoms of Dementia (BPSD)

Among the 589 subjects with MCI and early-stage AD, 247 (41.9%) subjects were classified as being well-nourished, and 342 (58.1%) were classified as being at risk of/with malnutrition. The differences in clinical profile between the well-nourished subjects and those at risk of/with malnutrition are shown in Table 3. While there was no difference in the MMSE scores between the two groups, those at risk of/with malnutrition showed significantly higher DBD scores; lower MNA-SF scores and BMI; lower performances in BADL, IADL, and vitality; higher GDS; and were associated with a higher prevalence of dyslipidemia than the well-nourished subjects. The serum levels of albumin and micronutrients were not different between the groups.

Table 4 shows the differences in DBD factor scores. With ANOVA, those at risk of/with malnutrition were shown to have higher factor scores in “verbal aggressiveness/emotional disinhibition”, “motor aggressiveness”, “behavior disturbance”, and “apathy/memory impairment” than the well-nourished subjects.

With ANCOVA conducted to control for confounding factors, Model 1 showed that nutritional status was significantly associated with “verbal aggressiveness/emotional disinhibition” (F = 14.95, *p* < 0.001), “motor aggressiveness” (F = 5.31, *p* = 0.022), “behavior disturbance” (F = 11.84, *p* = 0.001), and “apathy/memory impairment ” (F = 25.85, *p* < 0.001). Model 2 showed a significant association of nutritional status with “verbal aggressiveness/emotional disinhibition” (F = 5.87, *p* = 0.016) and “apathy/memory impairment” (F = 15.38, *p* < 0.001).

## 4. Discussion

The aims of this study were to clarify the nutritional status of patients with MCI and early-stage AD compared to NC, and to examine the relationship between nutritional status and BPSD. The present study demonstrated a higher prevalence of malnutrition in those with MCI and early-stage AD. Moreover, nutritional status was not associated with cognitive status, but it was significantly associated with BPSD, especially “verbal aggressiveness/emotional disinhibition” and “apathy/memory impairment” after adjustment for potential confounding factors. Nutritional status was associated with BPSD independently of cognitive impairment.

In the present study, patients with MCI or early-stage AD had lower MNA-SF scores and a higher prevalence of malnutrition than those with NC. Nutritional status as assessed by the MNA variances because of the differences between target populations [35]. A review of the literature showed that the prevalence of malnutrition as assessed by the MNA was 2% (0%–8%) among community-dwelling healthy elderly persons and 15% (8%–76%) among those with dementia [35]. Orsitto et al. [36] and Khater et al. [37] demonstrated that patients with MCI are more likely to have malnutrition than those with NC. In our study, the serum levels of albumin and folic acid were also lower in those with early-stage AD. The worsened biochemical markers of nutrition among patients with moderate dementia have been shown by a previous study [38], but we focused on more early stages of cognitive impairment. Based on these previous studies and our findings, those with cognitive impairment appear to have more nutritional problems than healthy elderly people, highlighting the fact that nutritional problems may start from MCI and early-stage AD.

The underlying mechanisms of weight loss as one of the major nutritional problems in AD patients have been accounted for by a decrease in energy intake, due to loss of appetite, as well as by an increase in energy expenditure, due to increased basal metabolism and behavioral disturbances such as wandering [39]. It has been reported, however, that the amount of physical activity and/or basal metabolism in AD patients is equal to or lower than that of those with NC [40,41]. In contrast, appetite loss and apathy leading to a decrease in dietary intake are observed with MCI and early-stage AD [4,5,42]. Further, in our study, those with MCI had lower scores for “food intake” and “weight loss” among the MNA-SF subscores than those with NC. In early-stage AD patients, “food intake” showed significantly lower scores, and “weight loss” was not statistically significantly different but tended to be lower (*p* = 0.046). Coupled with earlier reports, our study results suggest that weight loss in MCI and early-stage AD are likely to be associated with decreased food intake and appetite loss, although no information was obtained on the subjects’ actual food intake and eating behavior in this study.

One notable finding from this study is that the nutritional status of those with MCI and early-stage AD is significantly correlated with BPSD, particularly verbal aggressiveness, emotional disinhibition, apathy, and memory impairment. In general, it is considered that verbal aggressiveness and emotional disinhibition increase from moderate to severe dementia [43], and apathy and memory impairment increase from mild dementia. Among those with moderate to severe AD, the association between their nutritional problems and BPSD has been reported by some cross-sectional and longitudinal studies. Spaccavento et al. reported that some neuropsychiatric symptoms, including “aberrant motor behavior”, “apathy”, “hallucination”, and “nighttime disturbance”, were severe among AD patients with malnutrition than those without [16]. Guerin et al. investigated the longitudinal association of nutritional status with changes in cognition and BPSD, where nutritional status at baseline was shown to be associated with increased NPI (higher scores indicate worse behavior and psychiatric symptoms) scores after one year of follow-up (well-nourished subjects, +2.8; those at risk of malnutrition, −1.0; those with malnutrition, +10.0) [44]. In addition, White et al. investigated the association between neuropsychiatric symptoms and changes in weight over six months in AD, reporting that changes in NPI scores and weight were negatively correlated [17]. These previous studies indicate that nutritional problems influence the occurrence and severity of BPSD in AD patients. In this context, our study was the first to reveal a significant association of malnutrition with BPSD, even in those with MCI and early-stage AD. Since a recent review reported that individual nutrient and dietary patterns are associated with cognitive impairment [45], further studies investigating the association of nutrients and dietary patters with BPSD are also needed to confirm and deepen our findings.

The causal relationship between BPSD and nutritional problems might be complicated. As described above, some longitudinal studies showed possibility that nutritional problems increase individual BPSD. On the other hand, it is easy to imagine that some BPSD, such as apathy, leads to loss of appetite and inactivity, resulting in weight loss and nutritional problems in AD patients. Actually, some studies reported that apathy was a risk factor for weight loss in AD patients [39,46]. Although a causal relationship has not been elucidated, structural and functional changes in the brain have been suggested as the common underlying mechanism of a significant link between BPSD and nutritional problems. To date, some studies investigated the association between BPSD and brain changes using magnetic resonance imaging, single photon emission computed tomography, and positron emission tomography in patients with AD [47]. Ismail et al. [48] and Marshall et al. [49] showed that apathy and appetite changes were associated with hypofunction in the medial prefrontal area. Aggressive behavior, such as verbal aggressiveness and emotional disinhibition, which are shown to be related to nutritional status in the current study, has been shown to be associated with frontolimbic atrophy [50]. Moreover, cerebral small vessel disease, such as white matter hyperintensity (WMH), increases apathy [51]. Whereas, malnutrition is also shown to be related to these brain changes including atrophy of the medial temporal cortex [52] and increased WMH [53]. Furthermore, our previous study [54] showed that nutritional status positively correlates with regional cerebral glucose metabolism in the medial prefrontal areas in amyloid-β positive patients with MCI or early-stage AD. Thus, taken together, these studies appear to suggest that there may be structural and/or functional brain changes behind the association between nutritional problems and BPSD.

This study has several limitations. First, because of the cross-sectional design of this study, the cause-effect relationship between nutritional status and BPSD remains unclear. Second, because a previous study demonstrated gender differences in BPSD, our study focused on female patients alone. Therefore, our study findings may not be readily generalizable to the other sex. Third, the study suffered from a lack of information on other factors that could affect nutritional status, such as energy intake, appetite, and physical activity. Finally, while the domains of BPSD are often investigated by using the NPI, they were assessed by using the DBD scale. DBD scale does not include some important aspects of BPSD, such as delusions and hallucinations. Therefore, the association between nutritional status and more detailed symptoms of BPSD needs to be further explored.

## 5. Conclusions

In conclusion, the prevalence of malnutrition appeared to be increased in patients with MCI and early-stage AD. In addition, we found a significant association between nutritional status and BPSD, especially verbal aggressiveness, emotional disinhibition, apathy, and memory impairment, after adjusting for confounding factors. It should be noted that nutritional problems are potentially treatable, at least in part, and that attention should be given to preventing and caring for BPSD from MCI and early-stage AD onwards.

## Figures and Tables

**Table 1:** Factor analysis of Dementia Behavior Disturbance Scale subitems in subjects with MCI and early-stage AD.

	Verbal Aggressiveness/Emotional Disinhibition	Motor Aggressiveness	Behavior Disturbance	Apathy/Memory Impairment	Incontinence	Sexual Disinhibition
09. Is verbally abusive, curses	**0.683**	0.219	−0.018	−0.030	−0.138	−0.125
05. Makes unwarranted accusations	**0.652**	0.196	0.004	0.031	−0.189	−0.078
14. Moves arms or legs in a restless or agitated way	**0.576**	−0.075	0.038	−0.117	0.072	0.068
12. Refuses to be helped with personal care	**0.462**	−0.053	0.080	0.097	0.063	0.086
11. Cries or laughs inappropriately	**0.445**	−0.002	0.064	−0.043	0.033	0.043
13. Hoards things for no obvious reason	0.360	−0.138	0.031	0.308	0.047	0.027
15. Empties drawers or closets	0.344	0.003	0.041	0.111	0.046	0.070
19. Overeats	0.343	−0.074	0.097	0.010	0.251	−0.054
08. Repeats the same action over and over again	0.326	−0.099	0.193	0.094	0.096	−0.042
22. Makes physical attacks	0.062	**0.799**	−0.040	0.046	0.030	−0.042
23. Screams for no reason	0.106	**0.726**	−0.009	0.023	0.040	−0.068
26. Destroys property of clothing	−0.125	**0.509**	0.275	0.007	−0.006	0.064
28. Throws food	0.132	0.386	−0.135	−0.075	0.296	0.240
16. Wanders in the house at night	0.106	0.079	**0.667**	−0.143	−0.177	0.025
07. Paces up and down	0.189	−0.060	**0.550**	−0.016	−0.029	−0.042
04. Wakes up at night for no obvious reason	0.032	0.030	**0.512**	0.021	0.074	−0.077
21. Wanders aimlessly outside or in the house during the day	0.129	0.017	**0.466**	−0.080	−0.027	0.148
17. Gets lost outside	−0.265	0.386	**0.454**	0.054	−0.010	0.026
10. Dresses inappropriately	0.213	−0.105	0.365	0.074	0.153	0.005
18. Refuses to eat	0.042	0.049	0.244	0.126	−0.022	0.105
02. Loses, misplaces, or hides things	0.070	0.012	−0.119	**0.787**	−0.109	0.071
01. Asks the same question over and over again	−0.030	0.057	−0.078	**0.742**	−0.062	0.025
03. Shows lack of interest in daily activities	−0.055	0.045	0.119	**0.522**	0.108	−0.041
06. Sleeps excessively during the day	−0.061	0.035	0.225	0.233	0.188	−0.117
27. Is incontinent of feces	0.053	0.100	−0.165	−0.051	**0.790**	0.042
20. Is incontinent of urine	−0.055	0.016	0.065	−0.015	**0.696**	−0.119
24. Makes inappropriate sexual advances	0.000	0.018	0.084	−0.012	−0.027	**0.815**
25. Exposes himself/herself indecently	0.000	−0.042	−0.023	0.072	−0.071	**0.682**

Factor analysis, principal factor method and promax rotation. Items with significant loading (≥0.4) are shown in bold. (Cumulative contribution ratio, 51.9%). AD, Alzheimer’s disease; MCI, mild cognitive impairment.

**Table 2:** Clinical characteristics of subjects at the respective cognitive stages ^1^.

	NC (n = 152)	MCI (n = 271)	Early-Stage AD (n = 318)	*p* Value
Age, years	73.8 ± 5.2	76.4 ± 5.6 ^a^	78.3 ± 5.6 ^a,b^	<0.001
Education, years	11.5 ± 2.5	10.6 ± 2.0 ^a^	10.2 ± 2.2 ^a^	<0.001
Marital status, n (%)				
Never married	2 (1.3)	2 (0.7)	4 (1.3)	0.021
Married	106 (69.7)	159 (58.7)	171 (53.8)	
Divorced or widowed	44 (29.0)	110 (40.6)	143 (45.0)	
Living alone, n (%)	19 (12.5)	49 (18.1)	70 (22.0)	0.045
Body mass index, kg/m^2^	22.2 ± 3.3	22.1 ± 3.5	21.9 ± 3.3	0.588
MNA-SF, score	11.7 ± 1.9	11.1 ± 2.1 ^a^	10.7 ± 2.1 ^a^	<0.001
A. Food intake	1.95 ± 0.2	1.80 ± 0.4 ^a^	1.76 ± 0.5 ^a^	<0.001
B. Weight loss	2.37 ± 1.0	2.06 ± 1.0 ^a^	2.15 ± 1.0	0.004
C. Mobility	1.97 ± 0.2	1.98 ± 0.2	1.92 ± 0.3 ^b^	0.006
D. Psychological stress	1.62 ± 0.8	1.66 ± 0.8	1.56 ± 0.8	0.347
E. Neuropsychological disturbance	1.89 ± 0.4	1.75 ± 0.5 ^a^	1.55 ± 0.6 ^a,b^	<0.001
F. BMI	1.86 ± 1.1	1.82 ± 1.1	1.74 ± 1.1	0.588
MMSE, score	28.3 ± 1.9	25.1 ± 2.4 ^a^	23.2 ± 2.0 ^a,b^	<0.001
Barthel Index, score	99.2 ± 4.2	98.6 ± 4.9	97.0 ± 8.5 ^a^	0.001
Lawton Index, score	7.7 ± 0.7	7.3 ± 1.2 ^a^	6.2 ± 1.6 ^a,b^	<0.001
Vitality Index, score	9.8 ± 0.5	9.6 ± 0.7	9.2 ± 1.1 ^a,b^	<0.001
GDS-15, score	4.0 ± 3.0	4.6 ± 3.0	4.2 ± 2.8	0.049
DBD, score	5.7 ± 6.7	8.2 ± 6.7^a^	14.0 ± 8.9 ^a,b^	<0.001
Serum albumin, g/dl, n = 608	4.5 ± 0.2	4.5 ± 0.3	4.4 ± 0.3 ^a^	0.012
vitamin B_1_, ng/dl, n = 613	51.4 ± 48.3	46.1 ± 38.8	54.3 ± 82.6	0.132
vitamin B_12_, pg/dl, n = 610	753.1 ± 506.0	887.1 ± 1794.0	967.3 ± 4584.0	0.144
folate, ng/mL, n = 612	9.4 ± 3.9	9.2 ± 4.6	8.0 ± 5.3 ^a,b^	<0.001
Current smoker, n (%)	5 (3.3)	15 (5.5)	19 (6.0)	0.461
Drinking status, n (%)				
Never	113 (74.3)	212 (78.2)	258 (81.1)	0.315
ethanol <43.2 g/d	38 (25.0)	59 (21.8)	58 (18.2)	
ethanol ≥43.2 g/d	1 (0.7)	0 (0.0)	2 (0.6)	
Need for financial support, n (%)	8 (5.3)	11 (4.1)	21 (6.6)	0.394
Polypharmacy (≥5), n (%)	129 (47.4)	126 (46.5)	134 (42.1)	0.442
Comorbid conditions, mean	1.4 ± 1.1	1.5 ± 1.1	1.5 ± 1.0	0.879
Diabetes mellitus, n (%)	54 (35.5)	86 (31.7)	112 (35.2)	0.610
Hypertension, n (%)	69 (45.4)	162 (59.8)	190 (59.7)	0.006
Dyslipidemia, n (%)	73 (48.0)	123 (45.4)	133 (41.8)	0.412
Cardiac disease, n (%)	20 (13.2)	18 (6.6)	32 (10.1)	0.079
Stroke, n (%)	4 (2.6)	10 (3.7)	10 (3.1)	0.834

^1^ Values are means ± SDs unless otherwise indicated. AD, Alzheimer’s disease; BMI, body mass index; DBD, Dementia Behavior Disturbance Scale; GDS-15, 15-item Geriatric Depression Scale; MCI, mild cognitive impairment; MMSE, Mini-Mental State Examination; MNA-SF, Mini Nutritional Assessment-Short Form; and NC, normal cognition. *p* values are shown for differences at the respective cognitive stages. ^a^, *p* < 0.0167 post hoc comparison with NC. ^b^, *p* < 0.0167 post hoc comparison with MCI.

**Table 3:** Clinical characteristics of subjects with MCI and early-stage AD by nutritional status ^1^.

	Overall (n = 589)	Well-Nourished Subjects (n = 247)	Subjects at Risk of/with Malnutrition (n = 342)	*p* Value
Age, years	77.4 ± 5.7	76.9 ± 5.6	77.7 ± 5.6	0.098
Education, years	10.4 ± 2.2	10.5 ± 2.1	10.5 ± 2.1	0.581
Marital status, n (%)				
Never married	6 (1.0)	1 (0.4)	5 (1.5)	0.420
Married	330 (56.0)	137 (55.5)	193 (56.4)	
Divorced or widowed	253 (43.0)	109 (44.1)	144 (42.1)	
Living alone, n (%)	119 (20.2)	48 (19.4)	71 (20.8)	0.692
Body mass index, kg/m^2^	22.0 ± 3.4	23.6 ± 2.7	20.8 ± 3.4	<0.001
MNA-SF, score	10.9 ± 2.1	12.8 ± 0.8	9.5 ± 1.6	<0.001
MMSE, score	24.1 ± 2.4	24.2 ± 2.5	24.0 ± 2.3	0.488
Barthel Index, score	97.8 ± 7.1	98.9 ± 4.1	96.9 ± 8.5	0.002
Lawton Index, score	6.7 ± 1.5	7.0 ± 1.4	6.5 ± 1.6	<0.001
Vitality Index, score	9.4 ± 1.0	9.6 ± 0.8	9.3 ± 1.1	0.002
GDS-15, score	4.4 ± 2.9	4.0 ± 2.7	4.7 ± 3.0	0.013
DBD, score	11.4 ± 8.5	9.5 ± 7.3	12.7 ± 9.0	<0.001
Serum albumin, g/dl, n = 493	4.4 ± 0.3	4.4 ± 0.3	4.4 ± 0.3	0.522
vitamin B_1_, ng/dl, n = 497	50.7 ± 66.7	51.2 ± 69.6	50.3 ± 64.7	0.692
vitamin B_12_, pg/dl, n = 494	931.4 ± 3609.9	1205.4 ± 5558.8	745.0 ± 923.8	0.646
folate, ng/mL, n = 497	8.5 ± 5.0	8.3 ± 3.6	8.6 ± 5.8	0.544
Current smoker, n (%)	34 (5.8)	16 (6.5)	18 (5.3)	0.533
Drinking status, n (%)				
Never	470 (79.8)	196 (79.4)	274 (80.1)	0.452
ethanol <43.2 g/d	117 (19.9)	51 (20.6)	66 (19.3)	
ethanol ≥43.2 g/d	2 (0.3)	0 (0.0)	2 (0.6)	
Need for financial support, n (%)	32 (5.4)	14 (5.7)	18 (5.3)	0.831
Polypharmacy (≥5), n (%)	260 (44.1)	118 (47.8)	142 (41.5)	0.132
Comorbid conditions, mean	1.5 ± 1.1	1.6 ± 1.0	1.4 ± 1.1	0.093
Diabetes mellitus, n (%)	198 (33.6)	83 (33.6)	115 (33.6)	0.995
Hypertension, n (%)	352 (59.8)	157 (63.6)	195 (57.0)	0.110
Dyslipidemia, n (%)	256 (43.5)	122 (49.4)	134 (39.2)	0.014
Cardiac disease, n (%)	50 (8.5)	17 (6.9)	33 (9.6)	0.235
Stroke, n (%)	20 (3.4)	8 (3.2)	12 (3.5)	0.858

^1^ Values are means ± SDs unless otherwise indicated. AD, Alzheimer’s disease; DBD, Dementia Behavior Disturbance Scale; GDS-15, 15-item Geriatric Depression Scale; MCI, mild cognitive impairment; MMSE, Mini-Mental State Examination; and MNA-SF, Mini Nutritional Assessment-Short Form. *p* values are shown for differences between well-nourished subjects and those at risk of/with malnutrition.

**Table 4:** The association between nutritional status and factor scores of Dementia Behavior Disturbance Scale in subjects with MCI and early-stage AD according to ANOVA and ANCOVA.

	ANOVA				ANCOVA			
	Well-Nourished Subjects	Subjects at Risk of/with Malnutrition	F	P	Model 1 ^a^		Model 2 ^b^	
	Mean ± SD	Mean ± SD			F	P	F	P
Verbal aggressiveness/Emotional disinhibition	−0.17 ± 0.74	0.12 ± 1.00	15.36	<0.001	14.95	<0.001	5.87	0.016
Motor aggressiveness	−0.10 ± 0.48	0.07 ± 1.11	5.00	0.026	5.31	0.022	2.82	0.094
Behavior disturbance	−0.15 ± 0.62	0.11 ± 1.02	13.14	<0.001	11.84	0.001	3.18	0.075
Apathy/Memory impairment	−0.22 ± 0.85	0.16 ± 0.88	27.30	<0.001	25.85	<0.001	15.38	<0.001
Incontinence	−0.07 ± 0.89	0.05 ± 0.86	2.67	0.103	1.74	0.187	0.55	0.458
Sexual disinhibition	−0.00 ± 0.93	0.00 ± 0.85	0.09	0.913	0.01	0.920	0.15	0.901

AD, Alzheimer’s disease; ANCOVA, analysis of covariance; ANOVA, analysis of variance; MCI, mild cognitive impairment; and SD, standard deviation. ^a^ Adjusted for age and education. ^b^ Adjusted for age, education, marital status, living situation, Mini-Mental State Examination, Barthel Index, Lawton Index, Vitality Index, 15-item Geriatric Depression Scale, financial condition, polypharmacy, and comorbid conditions.
